# MD–Ligand–Receptor: A High-Performance Computing Tool for Characterizing Ligand–Receptor Binding Interactions in Molecular Dynamics Trajectories

**DOI:** 10.3390/ijms241411671

**Published:** 2023-07-19

**Authors:** Michele Pieroni, Francesco Madeddu, Jessica Di Martino, Manuel Arcieri, Valerio Parisi, Paolo Bottoni, Tiziana Castrignanò

**Affiliations:** 1 Department of Computer Science, “Sapienza” University of Rome, V. le Regina Elena 295, 00161 Rome, Italy; pieroni.1704202@studenti.uniroma1.it (M.P.); bottoni@di.uniroma1.it (P.B.); 2 Department of Ecological and Biological Sciences, Tuscia University, Viale dell’Università s.n.c., 01100 Viterbo, Italy; jessica.dimartino@unitus.it; 3 Department of Health Technology, Technical University of Denmark, Anker Engelunds Vej 101, 2800 Kongens Lyngby, Denmark; 4 Department of Physics, “Sapienza” University of Rome, P. le Aldo Moro, 5, 00185 Rome, Italy

**Keywords:** molecular dynamics, protein–ligand interactions, nucleic acid–ligand interactions, computational modeling of molecular systems

## Abstract

Molecular dynamics simulation is a widely employed computational technique for studying the dynamic behavior of molecular systems over time. By simulating macromolecular biological systems consisting of a drug, a receptor and a solvated environment with thousands of water molecules, MD allows for realistic ligand–receptor binding interactions (lrbi) to be studied. In this study, we present MD–ligand–receptor (MDLR), a state-of-the-art software designed to explore the intricate interactions between ligands and receptors over time using molecular dynamics trajectories. Unlike traditional static analysis tools, MDLR goes beyond simply taking a snapshot of ligand–receptor binding interactions (lrbi), uncovering long-lasting molecular interactions and predicting the time-dependent inhibitory activity of specific drugs. With MDLR, researchers can gain insights into the dynamic behavior of complex ligand–receptor systems. Our pipeline is optimized for high-performance computing, capable of efficiently processing vast molecular dynamics trajectories on multicore Linux servers or even multinode HPC clusters. In the latter case, MDLR allows the user to analyze large trajectories in a very short time. To facilitate the exploration and visualization of lrbi, we provide an intuitive Python notebook (Jupyter), which allows users to examine and interpret the results through various graphical representations.

## 1. Introduction

A detailed understanding of ligand–receptor interactions is crucial for the effective design of new drugs and for a general appreciation of cellular functioning mechanisms [1]. In this context, methods such as molecular docking and molecular dynamics, together with innovative machine learning techniques, are revolutionizing the approaches to the issue [2,3]. Molecular docking is a widely used tool for predicting and studying protein–ligand interactions [4,5,6]. This approach is also used as a starting point for the molecular dynamics simulation of the protein–ligand complex and the calculation of binding energy, providing valuable information about the structure of the complex and the strength of the interaction [7]. On the other hand, molecular dynamics provides a much more detailed picture of the temporal behavior of interacting molecules, enabling an investigation of not only stable conformations, but also the interaction path between the ligand and receptor [8]. In recent years, the usage of machine learning techniques, including neural networks and deep learning algorithms, has gained significant traction for the analysis of ligand–receptor interactions [9]. These approaches excel in identifying intricate and non-linear patterns within extensive datasets, rendering them highly suitable for analyzing omics data, such as the genome or proteome [10]. The integration of these advanced methodologies is paving the way for new frontiers in our comprehension of ligand–receptor interactions [11]. The profound impact of this convergence extends to enhancing our fundamental understanding of molecular interactions, accelerating the development of targeted therapies, and unlocking novel possibilities for advancing human health [12,13].

Molecular dynamics (MD) is a computational technique of great importance in the field of biological macromolecular systems, offering a means to simulate and study the dynamic behavior of such systems over time [14,15]. Studying ligand–receptor binding interactions (lrbi) with MD can provide insightful dynamical structural information about the macromolecular system and also offer valuable insights into the energy landscape of lrbi [16]. Understanding the intricate structure–function relationship of a ligand–receptor complex is fundamental to the fields of biochemistry and molecular biology. Furthermore, the identification of the key elements of lrbi is a fundamental step in guiding the drug discovery and design process [17]. The power of MD as a tool for analyzing ligand–receptor binding interactions provides insights into the complex behavior of these biological systems over time. MD analyses of such kind show broad applicability and success at every stage of modern drug design and discovery [18]. MD simulations provide a microscopic view of the motion and interactions of molecules, enabling the analysis of structural changes, energetics, and other properties that are critical for understanding the action of drugs. Running MD simulations, especially of large systems for extended periods, poses a significant computational challenge [19,20,21,22,23], and the hardware resources and storage required for such simulations are considerable [24,25]. This becomes even more evident when large simulation trajectories are generated, which can result in data files of tens or even hundreds of gigabytes in size [26,27,28]. Consequently, the downstream analysis of these MD simulations, which involves evaluating lrbi over time, can also be computationally demanding, in terms of both CPU usage and storage requirements.

In recent years, software tools optimized for high-performance computing (HPC) have been developed to handle large volumes of data in bioinformatics [29]. These tools leverage HPC clusters to process and analyze massive datasets efficiently, enabling comprehensive investigations into genomics [30,31], transcriptomics [32,33,34,35,36], proteomics [37], metagenomics [38,39], and structural bioinformatics [40,41,42]. In this last area, in order to address the computational challenges involved and to efficiently organize, manage, and analyze the lrbi generated by MD simulation over time, we introduce the MD–ligand–receptor software (MDLR), an HPC tool designed for analyzing MD trajectories of very large ligand–receptor systems studied over long time scales.

MDLR establishes a pipeline capable of delivering lrbi results in just a few hours. It achieves this by parallelizing the workflow on the available HPC resources. The software leverages two third-party applications: GROMACS [43], for the efficient extraction of conformational files (in PDB format) from the trajectory, and PLIP [44], for producing lrbi results for each PDB file. Subsequently, the pipeline stores the lrbi in a dedicated Python dictionary, where keys are tuples of the atom IDs involved in a specific lrbi.

One of the major highlights of MDLR is its adoption of a message passing interface (MPI) parallel implementation. This enables distributing each task across the available computational resources, significantly reducing the analysis time of the lrbi from days to just a few hours.

The software was built using Python (version 3.7) and employs the mpi4py Python parallel library (version 3.3) [45,46] for its parallel phase. The MDLR software is freely available on GitHub https://github.com/fraMade/MD_ligand_receptor (accessed on 16 April 2023) and Figshare https://figshare.com/articles/software/MD-ligand-receptor_software/23566245 (accessed on 16 April 2023). Instructions on its usage are provided in a comprehensive tutorial on the GitHub page, showcasing the command-line application. In addition, in order to enhance the user experience, an easy-to-use Plotly dashboard is provided, which allows users to visualize the pipeline results of lrbi through a variety of graphical visualizations.

## 2. Results

MDLR processes input derived from molecular dynamics simulations in the form of trajectory data. A trajectory is further divided into numerous discrete time intervals, designed to be examined concurrently. This parallel analysis facilitates efficient data processing. The goal of this computation is to efficiently generate a suite of data about lrbi, thereby illustrating the dynamic behavior of the ligand–receptor system over time.

In the following subsections, we present: (1) the main workflow of MDLR, (2) its software details, (3) a test case on the analysis of MD trajectory of a solvated system made of human DNA topoisomerase I in a complex with camptothecin venom (CPT) and in a covalent complex with a 22-base-pair DNA duplex, (4) some benchmarks showing the software performance in an HPC environment, and (5) the visualization tools developed to promptly plot the main features of lrbi, as an example.

### 2.1. MD–Ligand–Receptor Workflow

**Figure 1:**
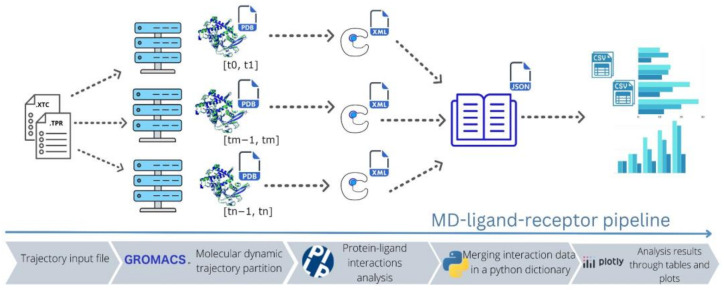
High-level description of the MD–ligand–receptor pipeline. Starting from the left, the software takes, as input, XTC and TPR files of the trajectory, which are then fed to a cluster for parallelization. The trajectory is partitioned in parallel in groups of PDB files, each corresponding to a specific time interval [tx,ty]. Then, PLIP analyzes each PDB group in parallel and extracts interaction data. Finally, results are merged and saved in JSON and CSV files, to be visualized with different kinds of plots.

The workflow of MDLR is depicted in Figure 1. The software accepts a trajectory from a molecular dynamics simulation as input and starts by segmenting it into numerous time intervals. These intervals can be examined in parallel, allowing for an efficient analysis process. The input data, therefore, comprise a trajectory file and an associated system topology file, both provided in XTC and TPR formats, respectively.

The workload is uniformly partitioned amongst the specified number of MPI processes. GROMACS software is employed by each of these processes to extract coordinate files, which are rendered in the Protein Data Bank (PDB) format. Each PDB file represents the state of the simulation at a specific temporal step. Each process employs the PLIP (protein–ligand interaction profiler, version 2.2.2) software to scrutinize the lrbi present within each respective PDB file. The significant information extracted from this analysis is subsequently encapsulated in XML files. Finally, all the data gathered by each process are consolidated into a single Python dictionary and are saved in both JSON and CSV formats. The aggregated data can then be visualized using the visualization tool proposed in Section 2.4.

### 2.2. MD–Ligand–Receptor Software Details

In this section, we delve into the intricacies of the components deployed in MDLR. The initial stage involves the activation of the MPI processes, to each of which an equitable portion of the workload is assigned via the MPI primitive scatter. This mechanism ensures an even distribution of the trajectory among all parallel workers. Given a molecular dynamics trajectory of length *T* and *p* MPI processes, the length of the assigned time interval for each process is then equal to *t′ = T/p*.

In the second stage of the pipeline, each parallel worker partition has assigned a chunk of trajectory corresponding to an interval of length *t′* (measured in picoseconds). The parallel worker extracts *n* configuration files in PDB format from the trajectory by launching, for each interval of length *t′*, the command trjconv of GROMACS. In standard cases, where trajectory configurations are saved every ten picoseconds, the number of extracted PDB files, *k*, is equal to *t′*/*10*. However, for significantly large trajectories, the size *t′* of the interval and, consequently, the number *k* of extracted PDB files, can increase. Hence, the amount of memory occupied by PDB files can become problematic. In order to solve this issue, each worker of MD–ligand–receptor extracts only a minimum fixed number of PDB files (default 10) at each iteration until the entire span *t′* is covered.

The third stage utilizes the PLIP (protein–ligand interaction profiler) to pinpoint the non-covalent interactions between the ligand and the receptor. This procedure is applied to each PDB file mined in the preceding step. PLIP provides all relevant information in an XML file, from which the lrbi data are subsequently extracted. Upon completion of this stage, all PDB and XML files are removed to liberate disk space.

In the final stage, once the time interval *t′* is completely covered, the corresponding extracted lrbi data are collected by a single process; this action is carried out by the MPI primitive gather.

All steps of the parallel pipeline are depicted in Algorithm 1, which presents the pseudocode for the algorithm. As a result, we obtained JSON and CSV files containing all the lrbi. Details of produced output files are listed in Table 1.
**Algorithm 1.** MD-ligand-receptor; The pseudocode describes the steps implemented in the pipeline.**Require:** (topology, trajectory);
Files describing the MD
**Require:** (start, end);


Positive time interval *T (ps)*
**Require:** time limit;


Set time limit for program execution1: **Procedure** MD-ligand-receptor(topology, trajectory, start, end, time limit)2: #Initial stage




3: comm = MPI.init()



# spawns MPI processes4: *rank* = comm.Get_rank()


 # gets MPI process id5: *t_start, t_end* = comm.Scatter(start, end)
# process obtains *t′* from time interval *T*6: N_PDB ← 100


    # fixes PDB number per iteration7: *interaction_table* ← dict()


8: *time* ← 0



9: *sub_t_start* ← *t_start* # initializes partial time interval *sub*_*t* for first iteration10:  *sub_t_end* ← *sub_t_start* + N_PDB


# sets end of *sub_t* interval
11:  #Second stage




12:  **while**
*time < time_limit* and *sub_t_start* ≤ *t_end*
**do**  # iterates until all of *t′* is covered13:  **if** *sub_t_end* > *t_end* **then**


14:    *sub_t_end* = *t_end*



15: **end if**



16: gmx_trjconv(*sub_t_start*, *sub_t_end*)
# splits the sub time interval in pdb files17: #Third stage



18: **for** *pdb_file* in directory **do**


19:  *plip_analysis* = PLIP(*pdb_file*)
# generates the xml file with interaction data20:  *interaction_table* = parse_xml(*plip_analysis*)
# extracts interactions from xml21:   **end for**



22:   remove(*pdb_file, plip_analysis*)
# deletes produced files to free disk space23:   *sub_t_start* += N_PDB

# updates *sub_t_start* for the next iteration24:    *sub_t_end* = *sub_t_start* + N_PDB

25:    *time* = timer()


26: **end while**



27: #Final stage



28: *interaction_table* = comm.Gather(*interaction_table*)

# gathers the final result29: **if** *rank* == 0 **then**
# the leader process merges all the interactions data30:    merge_tables(*interaction_table*)

31: **end if**



32: **end procedure**





Details about the programming language used and the parallel libraries employed can be found in Section 4.

### 2.3. Application to the Study of Lrbi for Human Topoisomerase 1 and Camptothecin

To demonstrate the capabilities of MDLR, we launched the pipeline on the system: human DNA topoisomerase I in a complex with camptothecin venom (CPT) and in a covalent complex with a 22-base-pair DNA duplex. The initial structure was downloaded from the Protein Data Bank (PDB ID: 1T8I [47]).

The biological system was chosen as the test case for MDLR, considering the pivotal role of CPT as a chemotherapeutic drug targeting human topoisomerase I (hTop1p) [48]. Human topoisomerase I is an essential enzyme involved in DNA relaxation, and its inhibition by CPT leads to DNA replication disruption and the activation of apoptotic response in cancer cells during the S phase of the cell cycle.

The reversible CPT inhibitor was initially extracted from the crystal structure and subsequently reinserted into the receptor. This was carried out in order to perform a molecular docking simulation, therefore providing us with the best pose (https://figshare.com/articles/dataset/MD-ligand-receptor_input_trajectory_best_pose/23566224, accessed on 16 April 2023), which was used as the starting structure for the molecular dynamics simulation.

**Figure 2:**
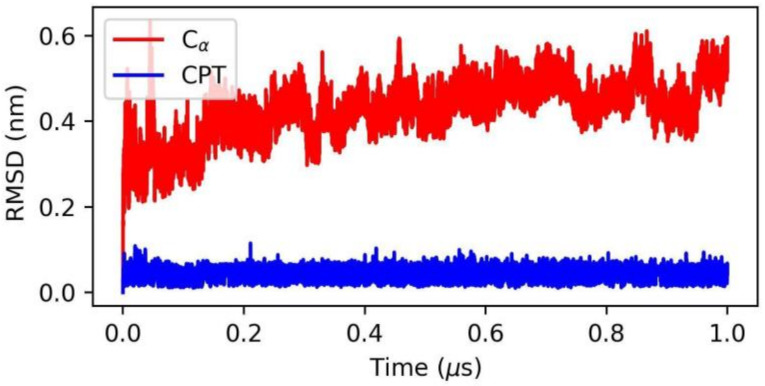
Root mean square deviation (RMSD) relative to 1 μs of simulation. The RMSD of Cα human topoisomerase 1 is shown in red, and the RMSD of compound CPT is shown in blue.

We conducted a molecular dynamics study of the system, immersed in a box of water molecules, to demonstrate the analytical capabilities of MDLR and its performance in an HPC environment. The system involved the addition of 137,340 atoms of water molecules, amounting to a total of 148,201 atoms. The classical MD simulation of 1T8I was carried out using the most favorable docked conformation of the CPT compound for a duration of 1 microsecond to study the overall stability of the receptor and CPT compound. The average root mean square deviation (RMSD) of both the human topoisomerase and the CPT compound, as a function of time, was observed to be, respectively, 0.43 nm and 0.05 nm (see Figure 2). This particularly suggests a high degree of binding stability of CPT, corroborating the observations made in the previous docking study (binding affinity: −8.5 kcal/mole) [26]. The trajectory resulting from the molecular dynamics simulation of this system (https://figshare.com/articles/dataset/MD-ligand-receptor_input_trajectory_test_case/23566080, accessed on 16 April 2023) was used as input for a test case analysis of MD–ligand–receptor. The results of the analysis, in terms of the generated outputs, are presented in in the following table.

**Figure 3:**
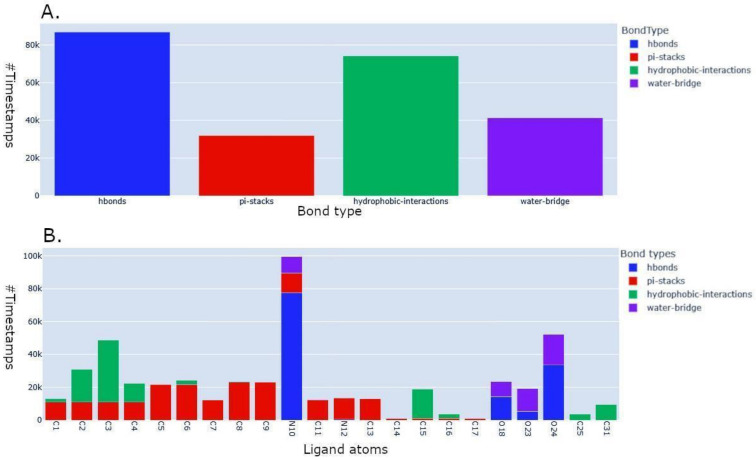
Plots of the bond types generated by the visualization tool after the analysis. (**A**): number of timestamps collected for each bond type. (**B**): detailed view showing the number of timestamps for each atom of the ligand, furtherly highlighted by bond type.

**Figure 4:**
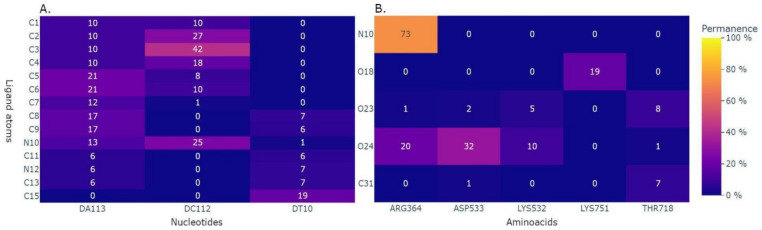
Permanence analysis among couples of ligand–receptor elements provided by the visualization tool. (**A**): percentage of interactions between each pair of ligand atoms and nucleotides. (**B**): percentage of interactions between each pair of ligand atoms and amino acids.

**Figure 5:**
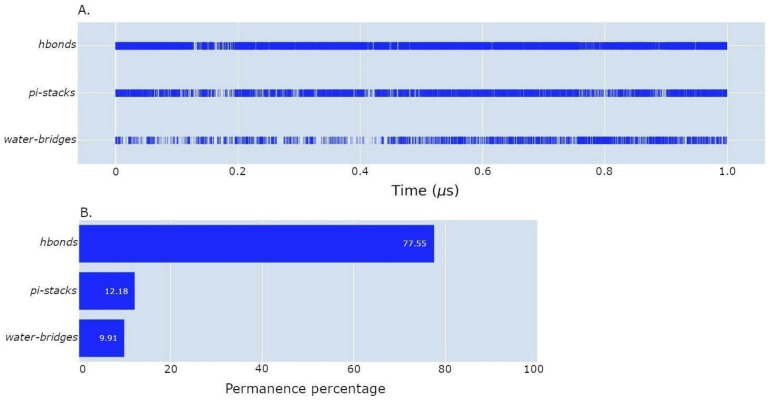
In-depth analysis of lrbi on different bond types provided by the visualization tool. (**A**): a histogram with a bar for each type of interaction performed. Each blue segment represents an interaction present at time t of the specific bond type. (**B**): a histogram that represents the permanence for each type of interaction in graph A. These plots are useful for understanding what type of interactions and when they are performed for a given ligand’s atom or a given couple of ligand’s atoms and a nucleotide or an amino acid receptor.

In the following, we will, respectively, describe the benchmark analysis to illustrate the software’s performance in an HPC environment and an additional tool for a quick visualization of the lrbi results. Figure 3, Figure 4 and Figure 5 depict several kinds of visualization of the results obtained from the analysis through the use of the Plotly dashboard described in the following Section 2.4. “MD–Ligand–Receptor Visualization Tools”.

The results of the application of MD–ligand–receptor to the human topoisomerase 1–camptothecin system were uploaded on figshare https://figshare.com/articles/dataset/MD-ligand-receptor_input_trajectory_results/23566230 (accessed on 16 April 2023). The zipped folder (md_cpt_test_case_results.zip) contains all the MD–ligand–receptor data results.

### 2.4. MD–Ligand–Receptor Benchmarks

In this section, we offer a comprehensive performance analysis of MDLR, with a focus on the elapsed time and attained speedup. As delineated in previous sections, the software provides the flexibility to initiate a preferred number of parallel processes. An increase in this number typically results in a decrease in execution time.

**Figure 6:**
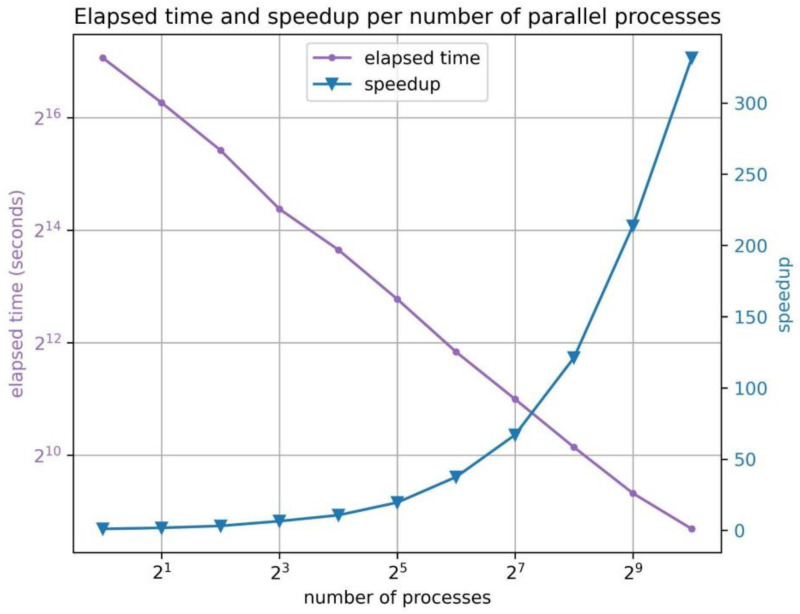
A depiction of the performance of the software. The *x*-axis presents the number of processes [1 → 1024] in a log scale, and the *y*-axis represents, on the left, the elapsed time in seconds on a log scale and, on the right, the speedup with regard to single process execution.

In order to assess the MDLR performance, we implemented an extensive series of tests utilizing a consistent trajectory input size of 0.1 microseconds (μs). We systematically doubled the quantity of processes, achieving a peak of 1024 processes. The results, visually represented in Figure 6 and summarized in Table 2, unequivocally demonstrate that the execution time is roughly halved when the number of processes is doubled. This finding highlights the substantial degree of independence among the processes, as both the trajectory partitioning and interaction analysis can be conducted without necessitating significant inter-process communication.

Throughout the execution, the only exchanged information consists of the initial workload assignment and the final analysis results, which were subsequently merged into the JSON file. These findings validate the efficiency and scalability of the software, emphasizing its ability to harness parallel processing power effectively.

### 2.5. MD–Ligand–Receptor Visualization Tools

We now present the visualization capabilities of MDLR, particularly focusing on its Plotly (https://plotly.com/python, accessed on 16 April 2023, version 5.15.0) dashboard, which offers a user-friendly interface for exploring the lrbi as a function of time. The dashboard produces a range of interactive plots that effectively capture the interactions performed by the ligand’s atoms and the receptor’s residues. This lightweight tool facilitates a prompt visualization of the lrbi results, enabling users to gain valuable insights into the dynamics of ligand–receptor complexes.

Firstly, the dashboard provides a histogram that showcases the overall count of lrbi for each kind of bond (an example of this graph is shown in Figure 3A). This visualization provides a comprehensive overview of the frequency of different types of interactions observed throughout the simulation. Additionally, a histogram is presented to display the permanence time of the interaction between the ligand and receptor for each ligand atom (an example of this graph is shown in Figure 3B). This histogram allows users to assess the duration of specific types of bonds, such as hydrogen bonds, pi stacks, hydrophobic interactions, and water bridges. To enhance visual clarity, each bar of the histogram is represented by a maximum of four colors, corresponding to the time of permanence for a particular bond type.

To further explore the lrbi, the dashboard provides informative heatmaps that visualize the percentage of interaction permanence between each ligand atom and individual protein residues and/or DNA nucleotides (examples of these graphs are shown in Figure 4A,B). The composition of the receptor can be customized to focus on protein-only, DNA-only, or protein–DNA interactions, allowing for a tailored analysis. These heatmaps play a crucial role in identifying key regions or residues involved in significant interactions, thereby enhancing our understanding of the structural dynamics within the ligand–receptor complex. Furthermore, the dashboard includes a plot depicting the time-dependent behavior of specific receptor residues and/or nucleotides during the trajectory. This plot, accompanied by an associated histogram illustrating the cumulative interaction time, facilitates a comprehensive temporal analysis of lrbi. These visualizations enable the identification of both stable and transient interactions, contributing to a deeper comprehension of the dynamic nature of the ligand–receptor complex.

Finally, the software offers a plot of the permanence of the interaction during the trajectory, focusing on a specific ligand atom and a designated receptor nucleotide or residue (an example of this graph is shown in Figure 5A). This visualization allows users to explore the dynamics of a particular interaction of interest, providing a detailed understanding of its temporal behavior.

To enhance the usability of the tool, the Plotly dashboard includes a dynamic filtering feature for each of the five described representations. This feature allows users to filter and visualize specific lrbi based on their preferences or research requirements, enabling a more targeted and customized analysis.

Overall, the visualization capabilities provided by the MD–ligand–receptor software empower researchers to gain comprehensive insights into the *lrbi* as a function of time. These visualizations aid in the identification of critical interactions, understanding the temporal dynamics, and facilitating the interpretation of the complex behavior exhibited by ligand–receptor complexes.

## 3. Software Comparison

### 3.1. Useful Tools for Preprocessing

MDLR was designed to natively analyze GROMACS trajectories. Accepting only one trajectory format as input, the Gromacs format, could be considered a limitation for MDLR. However, this can be resolved thanks to an additional easy step. GROMACS is one of the most popular software packages for molecular dynamics, along with others such as AMBER (version 23) [49], NAMD (version 2.14) [50], LAMMPS (version 15.06.2023) [51], and CHARMM (version c47b2) [52]. In general, the use of a specific software package depends largely on research needs, available hardware resources, and user familiarity with the software. In cases where a trajectory is not generated with GROMACS, but with other molecular dynamics tools, it is necessary to convert this trajectory into GROMACS format before using MDLR (version 1.0). To make it easier for users to use our pipeline with trajectories described in different formats, we report in Table 3 the list of trajectory conversion software toward the GROMACS format.

### 3.2. Comparing MDLR with Other Tools

Here, we introduce three software tools, JGromacs [57], MD-IFP [58], and a pipeline for visualizing large-scale protein–ligand interaction data sets (from here onwards, P1) [59], that can provide significant assistance for downstream analyses of molecular dynamics trajectories. For this reason, we highlight similarities and differences with MDLR.

JGromacs (version 1.0) is a general-purpose software for building additional analysis software, while MDLR is a pipeline that uses third-party programs, GROMACS, and PLIP to achieve a specific objective: the characterization and visualization of lrbi over time. Furthermore, MDLR enables an extremely rapid analysis through the use of parallel MPI libraries and does not require software development skills as it is an automated pipeline. Each of these components is important for highlighting the unique features of MDLR that make it powerful and versatile.

MD-IFP (version 1.1) provides an efficient computational engine for the estimation of the relative residence times of compounds against a macromolecular target and tools for obtaining insights into the underlying mechanisms determining ligand unbinding kinetics. This software has capabilities similar to PLIP for the study of lrbi and is able to automatically read trajectories in GROMACS format. Compared to MDLR, it is not optimized in terms of the calculation of lrbi over time and therefore is not able to exploit HPC resources. Furthermore, it does not provide incorporated tools for promptly visualizing the lrbi.

P1 provides an aggregated overview of the system. It focuses on the “contact” between the ligand and protein at different timeframes from a GROMACS trajectory, without providing specific details about the type of ligand–receptor interaction. It provides details of aggregated data in terms of mesh, i.e., a collection of vertices, edges, and faces that defines the shape of a polyhedral object in 2D/3D space, containing color values for each of the accumulated quantities. This software is optimized but conceptually different from MDLR results in terms of lrbi analysis and their molecular mechanisms.

In contrast, MDLR focuses on simulating and analyzing ligand–receptor systems using molecular dynamics. It offers a comprehensive analytical approach, providing detailed information on binding properties and system dynamics. Additionally, MDLR takes advantage of HPC resources, if available, for analyzing very large trajectories. Unlike the aforementioned software, MDLR integrates the PLIP software for an accurate analysis of protein–ligand interactions over time. In terms of visualization, MDLR is at the forefront and provides an intuitive user interface using Jupyter notebooks to create a dashboard based on the Plotly graphical visualization library. This simplifies the exploration and visualization of molecular dynamics simulation results.

## 4. Discussion

High-performance computing (HPC) resources are essential for an efficient simulation of molecular behavior in different environments, including aqueous solutions or vacuum conditions. By exploiting HPC, researchers can study the impact of various parameters on molecular behavior, leading to valuable insights into molecular interactions.

An important application of HPC resources is the study of molecular interactions, particularly in the context of drug design. HPC allows researchers to study the binding mechanisms between ligands and receptors, providing valuable information for the development of new drugs. With the help of tools such as MDLR, the structural dynamics of ligand–receptor complexes can be analyzed and visualized. This powerful tool efficiently handles large amounts of data and offers visualization capabilities to trace intricate details of the complex.

Among the specific advantages of MDLR, the software allows users to comprehend the behavior of a ligand–receptor binding system simulated realistically using the molecular dynamics (MD) technique. Hence, they can explore long-term binding interactions between ligands and receptors, obtaining detailed information on the binding properties and dynamics of the system. Furthermore, as mentioned above, MDLR is designed to run on HPC clusters, which enables it to handle large amounts of data and analyze complex systems efficiently. The software also offers an intuitive Python notebook-based user interface (Jupyter) to explore and visualize the results of molecular dynamics simulations.

However, MDLR requires a considerable amount of computational resources to analyze the molecular dynamics simulations, which may limit the size of the system that can be studied. Furthermore, the accuracy of molecular dynamics simulations depends on the quality of the force parameters used to describe the interactions between molecules, and the correct interpretation of the results produced requires some experience in analyzing molecular dynamics data. Finally, it is important to note that MDLR may not be able to accurately model some complex molecular interactions, such as protein–lipid or protein–carbohydrate interactions.

## 5. Materials and Methods

### 5.1. Software Included in the Pipeline

Two key software packages, GROMACS and PLIP (protein–ligand interaction profiler), are crucial components employed in our software pipeline.

GROMACS is a highly versatile and widely used molecular dynamics simulation package. It enabled us to simulate and analyze the dynamic behavior of the ligand–receptor system, facilitating several essential steps, including energy minimization using the steepest descent and conjugate gradient methods. Moreover, GROMACS performs molecular dynamics simulations in the isothermal–isobaric (NPT) ensemble, allowing us to balance pressure and temperature. These simulations provide valuable insights into the stability and conformational changes of the ligand–receptor complex over time. By solving Newton’s equations of motion, GROMACS accurately captures the intricate interactions and dynamics between the ligand and receptor, shedding light on the binding mechanism and intermolecular forces involved.

Another integral component of MDLR is PLIP (protein–ligand interaction profiler). PLIP plays a pivotal role in the downstream analysis of the ligand–receptor interactions. It efficiently extracts essential information from the MD trajectory, with a particular focus on the non-covalent interactions between the ligand and the receptor. PLIP’s robust functionality allows us to identify and analyze various types of interactions, including hydrogen bonds, hydrophobic interactions, pi stacking, and water bridges. By analyzing the resulting data, we have gained valuable insights into the key molecular interactions that contribute to the stability and binding affinity of the ligand–receptor complex. PLIP’s ability to generate informative visualizations, such as heatmaps and interaction plots, further facilitates the interpretation and understanding of the complex dynamics exhibited by the ligand–receptor system.

In summary, GROMACS and PLIP have played an indispensable role in the MDLR pipeline, allowing us to perform accurate molecular dynamics simulations and conduct in-depth analyses of ligand–receptor interactions. Their inclusion in our pipeline has ensured the robustness and reliability of our software in studying the dynamic behavior of molecular systems.

### 5.2. Programming Language and Parallel Libraries (MPI)

To develop MDLR, we used Python, a high-level programming language that is easy to use and is endowed with many ready-to-use libraries. mpi4py is a library that provides Python bindings for the message-passing interface, allowing applications to exploit multiple processors on workstations, clusters, and supercomputers. For software development and testing, we used version 3.1.3.

### 5.3. HPC Setup for Testing

To conduct all the previously described work, we used the HPC environment of CINECA. The organization allowed us to use the Galileo100 cluster to develop and test the proposed software. Galileo100 (https://wiki.u-gov.it/confluence/display/SCAIUS/UG3.3%3A+GALILEO100+UserGuide, accessed on 16 April 2023) is a supercomputer that uses the Slurm Workload Manager to schedule and execute typically parallel jobs on a set of requested nodes. The cluster is composed of 528 computing nodes having 2 × CPU Intel CascadeLake 8260, with 24 cores each, 2.4 GHz, and 384 GB RAM. This setup allowed us to easily test our software with a great number of available resources.

### 5.4. Input Data Preparation and HPC Environment

The pipeline starts by launching the command line, as shown in the GitHub tutorial https://github.com/fraMade/MD_ligand_receptor accessed on 16 April 2023 (version 1.0). For a reference to the workflow of the pipeline, see Figure 1. In particular, it takes as input the two result files of the molecular dynamics of the system: trajectory and topology (XTC, TPR) files produced with the GROMACS software. It is important for all the ligand’s atoms to belong to a single group, which has to be specified as an option in the input. The program accepts as parameters the number of parallel processes (N) and the start and end values (magnitude in ps) of the interval of the trajectory of interest. The software was developed in an HPC environment that uses Slurm as a scheduler. However, the software is also scheduler-independent and can be run on a simple server node. For further details, see the examples on the GitHub page.

### 5.5. Visualization Libraries

To develop the visualization tool, we employed the Plotly graphing library (version 5.9.0), which produces interactive plots. Furthermore, we used Plotly Dash, which, in combination with Plotly, produces dashboards with interactive plots.

### 5.6. Molecular Docking and Dynamics Protocol Utilized in the Test Case

As a first step, we downloaded the structure of human DNA topoisomerase I in complex with camptothecin venom (CPT) and in a covalent complex with a 22-base-pair DNA duplex from the Protein Data Bank https://www.rcsb.org (accessed on 10 April 2023) (PDB ID: 1T8I). The protocol used for preparing both ligand and receptor for molecular docking simulation is described in [60]. The structure was prepared using AutoDockTools [61] by removing the camptothecin, creating space in the receptor pocket for the subsequent docking with the ligand. We conducted molecular docking analyses launching Autodock Vina [62] on the CPT-deprived 1T8I complex as the receptor and CPT as the ligand. From the docking results, we selected the best pose of CPT, corresponding to −8.5 kcal/mole, as the starting structure for the molecular dynamics simulation of the solvated system. A classical molecular dynamics simulation was executed using GROMACS https://manual.gromacs.org/2021.2/index.html, accessed on 16 April 2023 (version 2021.2) [43,63] with the AMBER99 force field [64]. The topology of the CPT was generated using Acpype software (version 2022.7.21) [53] with AMBER atom types. The simulation complex, solvated with the TIP3 water model and neutralized with 18 sodium ions, underwent energy minimization using the steepest descent algorithm until the force fell below 1000.0 kJ/mol/nm. This was followed by two equilibration stages: first, a 1 ns NVT (number of particles, volume, and temperature) equilibration at a consistent 300 K temperature, and second, a 2 ns NPT (number of particles, pressure, and temperature) equilibration to stabilize the pressure at 1 bar. Long-range electrostatics were evaluated via the particle mesh Ewald method. Subsequently, unrestrained 1 us production simulations were performed with a 2 fs time step and coordinates were saved every 10 ps.

## Figures and Tables

**Table 1:** Names of MD–ligand–receptor output files with a brief description.

Output File	File Description
bonds_complete.json	JSON dictionary containing all the recorded interactions
hbonds.csv	CSV file containing all the recorded hbonds interactions
hydrophobic-interactions.csv	CSV file containing all the recorded hydrophobic interactions
pi-stacks.csv	CSV file containing all the recorded pi stacks interactions
water-bridge.csv	CSV file containing all the recorded water bridge interactions
salt-bridges.csv	CSV file containing all the recorded salt bridges interactions
LIG_ATOMS.csv	CSV file containing all ligand’s atoms
RCPT_ATOMS.csv	CSV file containing all receptor’s atoms

**Table 2:** Benchmarks for MDLR. Notably, an increase in the number of processes leads to an enhancement in speedup and a reduction in elapsed time.

# of Processes	1	2	4	8	16	32	64	128	256	512	1024
**Speedup**	1.0	1.7	3.1	6.4	10.6	19.5	37.5	67.0	121.2	213.7	331.6
**Elapsed time (s)**	136,948	78,788	43,857	21,266	12,874	7009	3654	2043	1130	641	413

**Table 3:** List of software for converting molecular dynamics trajectories into various formats. “Initial format” refers to the trajectory formats that can be read by the software, and “Final format” refers to the GROMACS conversion format. Some of these software packages also allow conversion to formats other than GROMACS, but the GROMACS format is the only one of interest for MDLR use.

Resource	Initial Format	Final Format	Link	Reference
ACPYPE	AMBER	GROMACS	https://alanwilter.github.io/acpype/ accessed on 16 April 2023, Version 2022.7.21	[53]
TopoGromacs	CHARMM	GROMACS	https://github.com/akohlmey/topotools/blob/master/topogromacs.tcl accessed on 16 April 2023, Version 1.8	[54]
InterMol	LAMMPS DESMOND	GROMACS	https://github.com/shirtsgroup/InterMol accessed on 16 April 2023, Version 0.1.2	[55]
MDTraj	NAMD TINKER DESMOND AMBER CHARMM LAMMPS	GROMACS	https://www.mdtraj.org/1.9.8.dev0/index.html accessed on 16 April 2023, Version 1.9.8.dev0	[56]

## Data Availability

Code: https://github.com/fraMade/MD_ligand_receptor, accessed on 16 April 2023 (version 1.0); Experiment data: https://figshare.com/articles/dataset/MD-Ligand-Receptor_a_HPC_tool_for_characterizing_ligand_receptor_binding_interactions_in_molecular_dynamics_trajectories/22591870/1, accessed on 16 April 2023.
